# Supplementary material for: Cryptic transmission of SARS-CoV-2 in Washington State
Source: medRxiv. 2020 Apr 16:2020.04.02.20051417. Preprint. [Version 2] doi: 10.1101/2020.04.02.20051417 (PMC7276023; doi:10.1101/2020.04.02.20051417)
Supplement: 1 [file NIHPP2020.04.02.20051417-supplement-1.pdf]

medRxiv preprint doi: https://doi.org/10.1101/2020.04.02.20051417.this version posted April 16, 2020. The copyright holder for this preprint (which was not certified by peer review) is the author/funder, who has granted medRxiv a license to display the preprint in perpetuity. This article is a US Government work. It is not subject to copyright under 17 USC 105 and is also made available for use under a CC0 license.

## Supplemental Figures

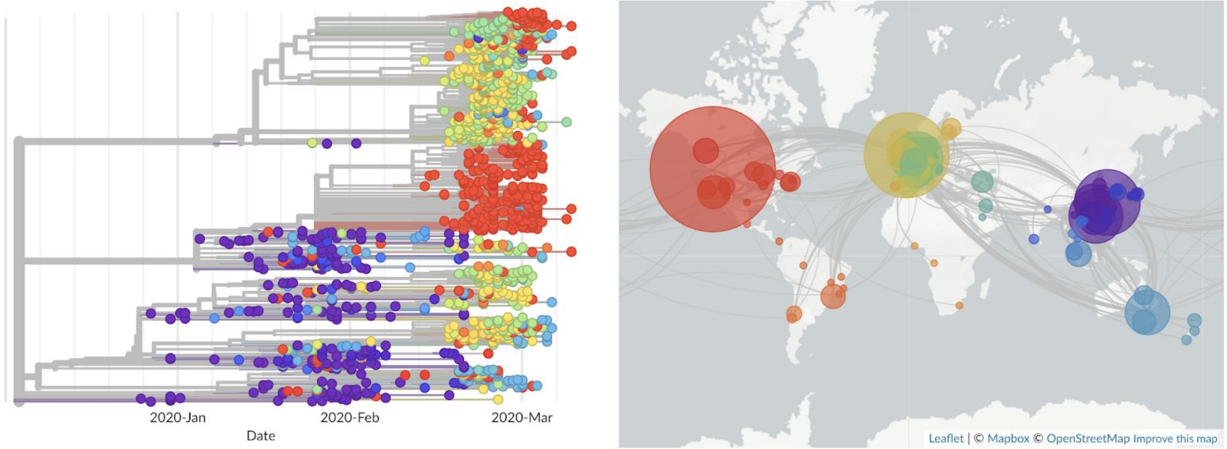


**Supplementary Fig. 1**. Phylogeny of 1442 SARS-CoV-2 viruses collected between December 2019 and March 2020 colored by country of sampling as shown in the map on the right. Viruses from China are shown in purple, viruses from Southeast Asia and Oceania in blue, viruses from Europe in yellow/green, viruses from South America in orange, and viruses from North America in red. Virus genome data shared through GISAID and phylogeny reconstructed by Nextstrain.
